# Report of Clinical Observations on Continued (Typhus and Typhoid) Fever

**Published:** 1850-09

**Authors:** Austin Flint

**Affiliations:** Prof. of the Principles and Practice of Medicine, and Clinical Medicine, in the University of Buffalo


### BUFFALO MEDICAL JOURNAL

AND

### MONTHLY REVIEW.

VOL. 6. SEPTEMBER, 1850. NO. 4.

### ORIGINAL COMMUNICATIONS.

ART. I.—*Report of Clinical Observations on Continued (Typhus and Typhoid) Fever. Based on an Analysis of Fifty-Two Cases.* By Austin Flint, M. D., Prof. of the Principles and Practice of Medicine, and Clinical Medicine, in the University of Buffalo.

(*Continued from page* 148.)

Section Third.—*Symptoms referable to the general aspect; Expression of Countenance; Decubitus.*—Characteristic changes wrought in the physiognomy, constitute an interesting branch of the symptomatic history of a disease. Such changes may involve various morbid effects. The physiognomy in health consists of several elements, the more important of which are the complexion, the facial muscles, and the eye. These elements, singly or collectively, being more or less modified, the general aspect, and the expression of countenance will be correspondingly altered.

In continued fever, some changes in physiognomy almost always occur; the changes, although not uniform, either in kind or degree, in some instances are striking, and in a measure distinctive.

Under the heading of this section, in the preliminary analysis, I have arranged whatever is recorded in the cases under examination. In several of the histories nothing is stated relative to this point. In these instances here, as with regard to other points, the inference is, that nothing unusual, or striking, was observed; but there probably existed more or less of that *dullness* or *listlessness* of expression, which is very rarely, if ever, entirely absent.

Directing inquiries to the elements of the physiognomy separately, the *complexion* comes first in order. In a large proportion of the cases, the complexion was altered, the face being *reddened*, sometimes of a *dusky* hue, and occasionally slightly *livid*. These changes were evidently due to capillary congestion. They were always more marked in the cheeks, than elsewhere, and sometimes were chiefly observable in that situation. This state of the capillary vessels, as will be perceived when we come to speak of the symptoms referable to the skin, in many cases extended, more or less, over the entire surface of the body, but in some instances it was exhibited on the face when it was not on any other part of the external tegument. It was always exhibited in the face, if present in other portions of the external surface.

I cannot suppose this symptom to have been peculiar to the cases that I have observed, and hence I am at a loss to understand why it has not attracted more notice than it appears to have done with clinical observers, more especially since, in connection with the humoral pathology of fever, it would seem to deserve consideration.*

The redness of the face resembled closely the appearance which the surface presents after exposure to cold, the explanation probably being the same in either instance, viz: retarded circulation of the blood in the capillary vessels. On pressure with the finger the redness disappears, and returns after the pressure is removed. The redness returned less quickly in proportion to its duskiness or lividity, showing that the color is an indication of the degree in which the forces of the circulation residing in the capillary system are depressed. This statement is made from recollection, but is believed to be correct.

The cases of the *Typhus* and *Typhoid* types present, on comparison, differences as respects this symptom. It was present in a large proportion of the *Typhus* cases. It is recorded present in *eleven* of the *twelve* hos-

* I do not mean to be understood by this remark that this symptom has escaped observation, but only that, so far as my knowledge extends, it has received less notice than would be expected from its presumed frequency, and pathological relations. Without having taken pains to consult authorities on the point, I cannot call to mind any writer who has alluded to it more distinctly than Dr. Gerhard, of Philadelphia. [See Graves and Gerhard's Clinical Medicine, page 733, and also Dr. Gerhard's account of Epidemic Typhus Fever in Philadelphia in 1836, published in the American Journal of Medical Sciences.] I do not discover that this symptom is embraced in the researches of Louis.

pital cases of this type, no mention being made of it in the single remaining case.

On the other hand, in the *eighteen Typhoid* cases it is recorded present in *ten*. In some of the remaining *seven* cases, its absence being stated, and in others no mention made either of its absence or presence. In none of the cases occurring in private practice is this symptom mentioned.

The two groups of cases differed also as to the hue of redness. In the *Typhus* group the hue was dingy or dusky, and in one case distinctly livid. This is noted in *five* of the *eleven* cases. In the *Typhoid* group, on the contrary, in *one* case only, is the redness recorded as dusky, and in *one* case somewhat dusky. As a general remark, the intensity of redness was greater, in a marked degree, in the cases of typhus than in those of the typhoid type, in those instances in which a dusky tint was not apparent. In most of the cases of the latter type, in which the symptom was present, it is stated to have been either moderate or slight. Such qualifications are not given in any of the cases of *Typhus* in which the symptom was present.

It may be suspected that the capillary congestion of the face and surface generally, is dependent on the complication of pulmonary disease, in consequence of which, the free transmission of blood through the lungs is prevented, and its æration compromised. With reference to this point, I have compared the pulmonary symptoms with the symptom now under consideration, in all the cases, and find that this connection does not uniformly exist. Several of the cases in which the congestive redness and dusky tint of the face were marked, presented pneumonic complication, denoted by cough and expectoration, accelerated respiration, and, occasionally, the physical signs of inflammation of the lungs, but these symptoms and signs were absent in other cases in which the appearances of the face were the same; and the lungs evinced notable disorder and inflammation in cases in which no capillary congestion is recorded. To speak with greater exactness of the cases of *Typhus*, congestive redness of the face, and marked disturbance of the respiration, were associated in *nine* cases. The congestive redness was present without notable disturbed respiration in two cases.

Of the cases of the *Typhoid* type, in but one case was congestive redness associated with marked disorder of the respiratory function, and in *two* cases in which pneumonitis existed, that symptom is not recorded as present.

Thus it appears that the condition of the capillary vessels is not to be attributed to a morbid condition of the lungs, or to derangement of the functional activity of the latter organs. It is worthy of note, however, that in *typhus*, in a large proportion of cases, capillary congestion is associated with pulmonary affection, while in *typhoid*, the absence of such a connection would seem to be the rule. It is highly probable that both pulmonary disorder and congestion of the surface, may be effects of the same prior morbid condition. It is not likely that capillary congestion in Continued Fever is limited to the surface, when it is present in the latter situation. Internal parts, could they be observed, might be expected to present similar appearances. The lungs, as is well ascertained, are liable to become congested during the febrile career, and the form of pneumonitis which usually occurs as a complication of fever, called *pseudo pneumonitis*, is usually supposed to result chiefly from passive engorgement of these organs. This complication, however, is thought to be far more liable to occur in *typhus* than in *typhoid* fever. The morbid condition then, whatever it be, which determines the congestive redness in the skin, is the occasion of congestions elsewhere, which may, or may not be sufficient in degree to produce a sensible physical change in the congested organs, leading to marked disturbance of their functions. In congestions, pervading the capillary system of the general circulation, the lungs are especially prone to become involved. This is probably the fact in cases of *typhoid* as well as *typhus*, in which congestive redness of the surface is observed, but not in a degree sufficient, generally, to lead to notable pulmonary complication, or marked disorder of the respiration. The latter consequences, it would be expected, should much oftener be present in *typhus*, inasmuch as in this type, not only is capillary congestion present in a much larger proportion of cases, but it exists in a much greater degree. This pathological reasoning is submitted as rationally consistent with the facts which preceded and suggested it.

And now the question can hardly fail to arise in the mind of the reader, upon what prior morbid condition *does* the state of capillary congestion depend. Is it dependent on the impairment of the nervo-muscular forces involved in the circulation of the blood, or is it incident to a morbid alteration in the blood itself? This question opens the way for a wide discussion, and would embrace topics of a speculative character. These I shall forego, as foreign to the historical plan of this memoir, and confine myself to the inquiry whether the cases in my collection can be made subservient to the developement of any farther results having a bearing on this inquiry.

If the congestion of the capillary vessels depends merely on the condition of the forces carrying on the general circulation, it would be expected that in those cases in which the former was marked, the pulse, the thermometer of the forces presiding over the movement of the blood through the heart and arteries, would denote a corresponding embarrassment of the latter. A comparison of the pulse in those cases in which capillary congestion was present, with those cases in which it was absent, is therefore desirable. It is not important to enumerate with respect to this point, but simply to ascertain if, in my collection of cases, the presence of capillary congestion is uniformly associated with symptoms denoting a degree of depression of the forces carrying on the general circulation, greater than when capillary congestion is not present. The symptoms by which a depressed or embarrassed circulation is to be estimated, relate to the pulse and the impulse of the heart. As regards the pulse, in proportion as it is frequent, feeble and irregular, the circulatory forces are to be regarded as compromised by the disease.

An examination of the cases with reference to this point, shows, that in much the larger proportion of cases in which capillary congestion is noted, the frequency and feebleness of the pulse were not greater than in cases in which no capillary congestion was observed. I counted *twenty-two* cases (including those of typhus, typhoid, and doubtful types,) in which capillary congestion was not associated with *unusual* disorder of the general circulation. In a few cases (*four*) this connection was found to exist; and in some cases, (*three*) there existed notable disturbance of the general circulation without congestive redness. In view of these results, is it not fair to arrive *par voie d'exclusion* at the inference that the source of the retarded circulation in the capillary system of vessels, in continued fever, lies in some morbid condition affecting these vessels, or their contents, more directly than through the intervention of the general circulation? That this morbid condition relates to the blood itself, rather than to the vessels, many considerations might be adduced to render probable, but this would lead us beyond the circle of our facts.

In connection with the pathological inquiries suggested by the symptom which has just been considered, it would be interesting and important to determine, to what extent, and under what circumstances, the symptom is present in other diseases than continued fever. This must be deferred for want of sufficient data for such a comparison.

I proceed now to consider elements of physiognomy other than the *complexion*. These are the *facial muscles*, and the *eye*.

The muscles of the face may present appearances representing the condition of the nervo-muscular forces generally, and they may also serve, to some extent, as indices of the mental state of the patient; in the latter point of view, however, they are generally to be taken in connection with the eye, and both together constituting the *expression* of the countenance.

As representing the nervo-muscular forces, the appearances which are found in the records of my cases are as follows: In *two* cases, *dropping of the lower jaw* is mentioned as a striking symptom. In both cases, the disease, judged by other symptoms, exhibited great severity, and in one of the cases it proved fatal. In the latter case, the symptom was associated with extreme prostration, with sliding down in bed. In the case that remains, the muscular prostration was very great.

*Tremulousness of the muscles of the face,* as if from mental emotions, was another symptom noted as present in *two* cases. In one of these cases, the disease proved fatal. In this case the symptom was much more marked, and was associated and tremulousness of the hands, and, at times, inability to protrude the tongue.

*Abolition of the expression peculiar to the individual*, was, of course, present in the fatal cases, as it generally is in different diseases, toward the close of life, entering into what is known as the Hippocratic countenance.

In speaking of the two preceding symptoms, i. e., depression of the lower jaw, and tremulousness of the facial muscles, it is not to be understood that they were present only during the last moments, for, at this period, they are not unusual, but during the career of the fever, prior to the last stage.

The *expression* in the greater number of the cases is described as *dull, heavy, stupid, vacant,* terms expressing different degrees of the same general aspect. This physiognomical appearance, in a large proportion of fever cases, is striking, and, in a measure, pathognomonic. There is a lack of expression, a deficiency, not only of animation, but intelligence, a look of stupidity and indifference, which, to the practiced observer, often suffices to reveal, at a glance, the character of the disease. This general rule, however, is not without exceptions. In *one* of my cases, the habitual expression was that of mirthfulness, and in another case, the patient was cheerful and generally smiling. In another case, the countenance during the whole career of the fever wore a marked lugubrious expression. These appearances are certainly rare. In the great majority of cases, patients with continued fever manifest by the countenance, or in other ways, neither agreeable nor painful emotions.

The only peculiarities which I have noted, not included in the foregoing summary, are, an expression like that of a person confused, and attempting to collect his ideas; an expression of astonishment; an expression of wildness, the latter being associated with active delirium.

The *decubitus* is a point upon which the records of the case, for the most part, are silent. In a few of the cases, it is stated that the patients lay on the back, and generally, in connection, the tendency to slide downward in bed is noticed. These were invariably cases of great severity of disease, attended by extreme muscular prostration. In other instances, it is mentioned that patients voluntarily assumed a posture on the side, as an indication either of mildness in the disease, or abatement of its severity. All who have been accustomed to observe cases of this affection, have noticed that, as a general remark, the patient lies on the back, in proportion to the gravity of the disease, maintaining the same position, seldom, or never changing it of his own accord; and in cases of extreme prostration, the body yields to the force of gravitation, and, the head and trunk usually being elevated, tends constantly to slide downward, so that unless prevented by a foot board, or by the attentions of those around him, the feet will be found sometimes to protrude beyond the bed.

### SECTION FOURTH.

*Symptoms referable to the Nervous System. Mind. Sleep. Coma. Senses, and Sensibility. Muscular Contractions, etc.*

The *nervous system*, in Continued Fever, is almost invariably, if not uniformly, the seat of some morbid phenomena. So constantly is this the case, that, as the reader need not be informed, a doctrine of the pathology of the disease, which has many supporters, if, indeed, it be not still the commonly accredited theory, attributes the primary and essential morbid changes, constituting, *par excellence*, the disease, to this portion of the organism. The symptoms referable to the nervous system are various in kind, as well as in degree. Some of the more prominent of the several sources of symptomatic events are enumerated in the heading to this Section, and I will proceed to consider them under separate divisions.

*Mind.* Under this division, the subject of *delirium* first suggests itself. In much the larger portion of instances, more or less delirium is manifested during the febrile career, but cases are far from exhibiting uniformity with respect to this symptom. The delirium is sometimes *active*, that is to say, the patient requires to be closely watched, and, possibly, to be placed under physical restraint. Oftener it is *passive* in its character, denoted by incoherent conversation, and muttering. In the lightest shades of mental aberration, the patient occasionally forgets where he is, and the circumstances of his illness, but recovers himself in a few moments. This transient aberration is most apt to occur on first awakening from sleep.

What is the relative frequency in occurrence of the two varieties of delirium just mentioned, viz., *active* and *passive* delirium; and what relations does each kind have to the different types of fever, to the issue of the disease, and to associated symptoms? These are the inquiries which are to direct the analysis of the data before us.

Of the *twelve* cases of *Typhoid* fever in *private practice*, the delirium might be pronounced active in *one* case. Passive delirium, more or less in degree, existed in *eight* cases, and no delirium was exhibited in *three* cases. In the single case of *Typhus* in this group, the delirium was not active; it was active in the single case of *doubtful type.*

In the *hospital cases*, of the *eighteen* cases of *Typhoid*, *active* delirium was present in *five*, the delirium was *passive* in *nine*, and *four* cases exhibited no delirium.

Of the *twelve* cases of *Typhus*, not *one* manifested *active* delirium, the delirium was *passive* in *eleven* cases, and no delirium was exhibited in *one* case.

Of the *eight* cases of doubtful type, *passive* delirium existed in *seven*, and no delirium was apparent in *one* case.

The above enumerations show that the existence of more or less delirium is the rule in continued fever of either type, but that exceptions to the rule occur in both types; that this rule obtains more generally in *Typhus* than in *Typhoid*, and it is chiefly in cases of the latter type that the delirium is active in its character. Should the latter point be established by a sufficiently large number of observations, it may sometimes be available for diagnosis, as respects the two types, at a period of the disease before other distinctive features have become developed.

In order that the above results may be appreciated, the principles upon which the distinction between *passive* and *active* delirium has been made, requires some explanation. As in every instance in which a division is based chiefly on difference in degree, the dividing line is not easily fixed, and must be determined by a rule somewhat arbitrary. Between a slight shade of aberration, and an excess of activity of delirium, there is a wide interval; but this is not the case at the point where the two extremes approximate; in other words, it is difficult to say precisely what amount of delirium ceases to be passive, and becomes active. The course I have pursued, practically, as nearly as I can define it, is this—when the incoherency has been boisterous, manifested by much and loud talking, shouting, etc., I have considered the delirium *active*, although the patient may not have required coercion. On the other hand, occasional, or even frequent efforts to get up, if the patient be at once persuaded to lie down, or to return to his bed, have not been sufficient to constitute *active* delirium, but the term has been restricted in its application to cases in which such efforts have been not only frequent, but perseveringly repeated, rendering constant supervision, or even mechanical restraint necessary.

The characters which belong, in general, to this symptom, as it is presented in continued fever, and its diversities in the different cases under examination, claim further notice than merely to distinguish them as active and passive. I will therefore devote some space to these points, without taking pains to obtain numerical results.

In the majority of cases in which passive delirium existed, the manifestations consisted in the patients talking to themselves, muttering, asking questions which were irrelevant, or inconsistent with their situation, frequently relating to business affairs, declaring that they were well, and desirous to go home, and in attempting to get out of bed. The effort to get out of bed, seemed sometimes like sleep-walking, and when patients assigned any motive, it generally differed at every separate attempt. The request to lie down was usually readily complied with, and was followed by quietude for a time, when the effort would perhaps again be repeated, and so successively, with intervals of greater or less duration. It happened several times, when patients had not given any evidences of mental aberration which seemed to require them to be carefully watched, that they succeeded in getting out of bed unobserved, and in partially, or entirely dressing themselves. An attendant is liable to be deceived by the representations of the patient, and suppose that it is proper to allow him to get up. For example, in one of the cases, on the third day, before the diagnosis had been positively made, I found the patient, at the time of my visit, sitting up, entirely dressed, saying he was quite well, that his mother had just arrived in town, and he was going out to see her. On being desired to undress and go to bed, he complied without the least reluctance. The attendant had supposed him to be as much better as he professed to be, and had not interdicted his dressing, which he was able to accomplish, not being, at that stage of the disease, greatly prostrated.

In none of the cases does there appear to have been a persistent delusion, giving a fixed subject and purpose to the conversation or movements, but on the contrary, it would seem that a constant succession of incongruous ideas characterizes the delirium of continued fever.

The delirium was observed invariably to be greater during the night, than in the day time. In some cases, the only manifestations occurred at night, the patient apparently being rational during the day.

In *four* instances, paroxysms of weeping and sobbing occurred, as in some forms of hysteria, the patients being all of the male sex. In two of these cases, the disease proved fatal, and the paroxysm occurred, in each, at the latter part of the disease. In both these cases, the cause assigned for grief was, that the patient had committed some heinous fault which would be followed by disgrace. In each of the two cases in which recovery took place, the weeping paroxysm occurred near the period of convalescence. In one of these cases, the source of grief seemed to be apprehension of death; in the other case, the patient said he would not tell why he wept. This variety of mental aberration presents a striking contrast with the kind of delirium usually present in continued fever, which does not involve any acknowledgment, or apparent consciousness, either of bodily or mental distress.

A trait somewhat peculiar to the mental condition in fever, is that the patient generally replies to questions rationally. Even in cases in which spontaneous incoherency is marked, when the attention is directed by a question, the mind appears to be capable of rational action, but immediately afterward, perhaps, relapses into its former dreamy state.

It does not follow, however, because replies to questions are consistent, that they are therefore correct. This is far from being the case. The statements given by patients with fever are often erroneous, when their intelligence appears perfect; and, hence, they are not to be relied upon for information of the previous history, or the daily events of their illness.

We are apt, also, to be deceived in estimating the mental condition of fever patients by the absence of manifestations of delirium. In some of the cases that I have observed, I had supposed, during the progress of the disease, that the intelligence was in no degree compromised, when, after recovery, patients have assured me that they had a very imperfect recollection of what had transpired. For example, in a case occurring in private practice, the patient had been attended by a homœopathic practitioner for several days, until, the friends becoming dissatisfied, he was asked if he did not wish another physician. He requested that I should be sent for, and daily saluted me by name, replied to questions coherently, giving no marked indications of delirium prior to his convalescence, but he afterward informed me that he had no recollection whatever of desiring my attendance, or of the fact that I visited him while the febrile career continued. The only explanation he could give of his electing my services was, that he had been accustomed to see me at church.

Of the few cases in which the delirium was *active*, it was remarkably violent in *two*. In both these cases, the efforts to get out of bed were almost incessant, requiring constant vigilance and considerable restraining force on the part of the attendants. These delirious effects continued up to a few hours of dissolution, both cases terminating fatally. In each of these cases, the type of the disease was *typhoid*.

Cases in which such activity of delirium is a prominent feature, may be considered and treated as cases of *encephalitis* by those who are not thoroughly versed in the principles of diagnosis. The early history of the disease, the state of the senses, the presence of abdominal and the symptoms so often associated with fever, etc., should, in most instances, furnish sufficient data for a correct discrimination.

Cases, however, do occur, in which, owing to defective information respecting the duration of the disease, and the previous history; to peculiarities in the cerebral symptoms, and the absence of many of the distinctive characters of fever, the diagnosis presents great difficulty. The following case will serve to illustrate this remark, and at the same time, furnish an instance of an unusual variety of mental aberraation:

Frederick Brighton, German, aged 13, entered hospital September 9th, 1849. He was incompetent to give any account of his illness. From what was said by his sister, who brought him to the hospital, I gathered that he was attacked five days before his entrance, with diarrhœa and vomiting. His mother was buried on that day, having died with epidemic cholera. He had been delirious from the commencement. Had had no recurrence of diarrhœa or vomiting since his attack. It was not ascertained that he had had any medical attendance. He was actively delirious during the night after his admission; and in the morning succeeded in making his way to the verandah communicating with the ward, and endeavored to jump over the railing. At my visit he was somnolent, easily roused, but not to complete intelligence. Keeps his mouth in motion during sleep, as if sucking. (This symptom I have observed in another case.) Skin is cold and dry. Pulse 92. Tongue, which is readily and fully protruded, is moist and furred. Says he has no pain. No dejection since his entrance, nor has he passed urine.

*Sept.* 11. Somnolent. Respiration heavy, with loud whistling nasal rale, numbering ten. Actively delirious during night, endeavoring to get out of bed. Easily roused so as to open his eyes, but lies with a vacant stare, respiration continuing the same. Eyes not injected; pupil neither contracted nor dilated. Skin cool. Pulse 94, and somewhat thrilling. Flies creep over the face without exciting uneasiness. Commences to reply to questions, but fails to finish sentences. Protrudes his tongue, which is covered with a white coating; indentations of the teeth on the side of the tongue. No dejection. Abdomen not distended. Several dark petechial spots on abdomen. Resists examination of the abdomen, apparently from disinclination to be annoyed. Passed urine freely yesterday afternoon.

12th. Somnolent. Respiration 12. No dejection. Urinates in bed. Active delirium at night. Skin cool. Pulse 112. Protrudes his tongue readily, which is moist and coated. Resists examination of the abdomen. Partially roused without difficulty, opening his eyes in part only, with an absence of expression.

*Treatment.*—Tart. ant. and pot. gr. ss. hourly. Head to be shaved and vesicated.

13th. Symptoms not materially altered. Pupils appear dilated, and do not contract on opening the eye-lids. Grinds his teeth loudly. The antimony was repeated yesterday several times without effect. One grain was given to-day with no effect. On attempting to make him protrude his tongue, (which was ineffectual,) he uttered a loud and piercing shriek. No dejection.

Five drops of croton oil were given on this day with no effect, and an enema containing two drachms of the spirits of turpentine.

14th. Died at one, A. M.

*Autopsy* — *Head.*—Moderate congestion of brain. No effusion into verticles. Some effusion into arachnoid sac, but quantity not ascertained, owing to escape of blood into this cavity. Consistence of brain normal. No exudation of fibrin, or opacity of arachnoid. No abnormal consistency of cerebral substance discovered.

*Abdomen.*—Small intestines contained a thin, yellowish substance. Colon much distended with gas. Peyer's glands much hypertrophied without ulceration; and corresponding mesenteric bodies greatly enlarged. No lumbrici. Stomach presented several patches of ecchymosis; mucous tunic not softened, but detached with unusual facility. Spleen small and not softened. Liver presented nothing worthy of note.

To some of my readers it may appear censurable not to have recognized the disease in this case by the symptoms, but I am bound to confess that the diagnosis was not made prior to the autopsy.

In another case (which occurred in private practice,) the delirium was of an unusual and striking character. I saw the patient, in consultation, on the seventh day of the disease. He had previously manifested passive delirium at night, but had seemed rational in the day time. He was greatly prostrated at the time of my visit, and the general symptoms denoted that the case would soon terminate fatally. In the examination of the case, I proceeded to ask several questions, to which he made no reply, but regarded me with a fixed, vacant stare. Suddenly he shouted with a loud and violent tone, "G——d d——n you, I'll kill you!" This was uttered with a piercing voice, and an expression of countenance which combined the utmost imaginable degree of terror and fierceness, rendering the language more startling and terrific than can well be described. Shortly afterward, he became calm, and replied coherently to questions, but before I left the house, he had two other paroxysms, in which, from expressions uttered, he appeared to imagine he was struggling in a mortal conflict. Although his muscular strength was so much reduced that he was unable to rise from the bed, he shouted so loudly as to be heard over the whole house, and even at a distance from it. The case terminated fatally the following day.

This patient had always been regarded as a man of desperate character, capable of almost any act of violence, and was supposed to have committed heinous crimes. The peculiar kind of delirium seemed to correspond with the character of the individual; and the inquiry suggests itself, may not the delirium of fever be influenced, in some instances, to a greater or less extent, by the habits, occupation, and moral constitution of the patient?

What relations does delirium sustain to the *issue* of continued fever?

Delirium existed in all the fatal cases in my collection. In each of the fatal cases of *typhus*, it was (of course) passive in its character. In the fatal cases of the *typhoid* type, it was active in three cases, and passive in three cases. The three cases, however, in which the greatest degree of activity of the delirium was manifested, all proved fatal. In the fatal cases of *doubtful type*, the delirium in one was active, and in the other passive.

In so far as any deductions from so limited a number of observations are admissible, the conclusions are, that although an unfavorable prognosis is not to be predicated on the fact that delirium is present in a degree sufficient to be pronounced active, yet such cases are more likely to prove fatal, and that an unusual violence of delirium should lead us to anticipate a fatal result.

It remains to propose another inquiry, viz: upon what pathological condition or conditions, is delirium dependent; in other words, what is the pathological explanation of the delirium in continued fever? This, as well as other questions, which, in the present state of knowledge, are open for discussion, and involve considerations more or less speculative, I shall only notice, in so far as facts can be made to bear upon it. With reference to the inquiry, whether this symptom may not be attributable to some one or more of the events appertaining to the febrile career, the method suggests itself of comparing the cases with each other to ascertain whether delirium is uniformly associated with any other particular symptom, or group of symptoms. But the symptom under consideration, being present in the great majority of cases, is apparently not influenced by the varied events which enter into the history of continued fever. It would seem that it must depend on something incident to that morbid condition which constitutes the essential nature of the disease. In all the cases in which delirium was not exhibited, the fever was of a mild grade, and it has been seen that it was not absent in a single case of those which proved fatal. Hence, it would follow, that the symptom depends somewhat on the severity of the disease; a conclusion which accords with the supposition that it proceeds directly from the proximate cause of the fever, and is not occasioned by any of the circumstances subordinate to the disease. In reasoning thus, it is assumed that the delirium of fever does not imply the existence of inflammation of any of the encephalic structures. That it does not, is rendered sufficiently plain by the symptoms, unless it be imagined that inflammation of these structures complicating fever, is deprived of all its diagnostic features except delirium, and this symptom we know does often occur, in other than fever cases, without involving inflammation. But positive observations have established that delirium may exist in fever, in a very active degree, without encephalic inflammation. This was the fact in two cases proving fatal, of those under analysis, in which active delirium was present. The autopsies revealed no evidences of inflammation within the cranium. In one of these cases the delirium was a more prominent and persistent symptom than in any other case that I have observed.*

Lesions of the mucous tunic of the stomach existed in both of the cases just referred to, consisting, in one case, in patches of ecchymosis, and, in the other case, in ulcerations. The observations of Louis, however, show that active delirium may exist in fever, irrespective of appreciable evidences of gastric disease.

There are several points of inquiry, relating to the subject of delirium, which have not been noticed. Among these are the duration of this symptom, and the period in the career of the disease when it first appears. The latter is a point involved in the discrimination of the two types of fever from each other. In the *typhoid* type it is stated that delirium occurs later than in *typhus*. On each of these points the data are too defective to be of much value. This is owing, in part, to the difficulty of fixing the date of the commencement of the disease in many cases, and, in part, to omitting, in the preliminary analysis, to note the time when the symptom first appeared, and the period of its continuance. To supply this omission, it would be necessary to go through with a re-perusal of all the cases. In examining the facts as arranged, with reference to the points mentioned, I find that in *four* cases of *typhus*, and *seven* cases of *typhoid*, delirium commenced early in the disease, i. e. during the first week; but it is not to be inferred that the same might not have been true of more or less of the cases in which evidence of the fact does not appear.

In a considerable number of cases, I find it stated that delirium was exhibited early in the disease, and diminished, or disappeared for some days before the career of the fever was ended.

The manifestations of a morbid state of mind, irrespective of delirium, deserve some attention. I have copied all that is contained in the preliminary analysis relating to this subject, and will give the expressions made use of in the records to denote apparent mental conditions of the patients, stating relative proportions of cases in which they respectively occurred, in general terms, without taking pains to obtain exact numeral results. *Dullness* of the intellectual operations was the trait most frequently noted in the cases of either type of fever. The patients replied to questions after more or less delay, seeming as if lost in deliberation, or as if the mind acted

*The case referred to was reported for the Buffalo Medical Journal Vol. IV., page 487, No. for January, 1849.

very slowly; when not addressed, they took little or no notice of persons and objects around them, seldom asking for any thing, expressing no wishes, appearing indifferent, and sometimes stolid. This was the general rule, but with some exceptions. In a few cases it is stated that the patients replied to questions promptly, and the apprehension had, apparently, lost but little of its normal quickness. In by far the greater proportion of cases, no emotions, either of pleasure or unhappiness, were exhibited. To this rule there were also exceptions. In one case, the patient was much affected at being ill at the hospital, away from his friends, and shed tears; in another case, great peevishness existed, the patient constantly complaining and whining; in another case, the patient was disposed to complain, and was apprehensive as to the result of the disease. Impatience at being questioned, apparent reluctance to reply, and sullenness, were occasionally observed; so also irritability of temper, which was an exceptional trait. These remarks refer to the mental conditions after the fever became established, and during its career. They do not embrace the period of access. In two instances, one of the typhus, and the other of the typhoid type, there was a disposition to indulge in hilarity and playfulness, as if under moderate exhilaration from an opiate, or alcoholic stimulus, and in these cases the approach of convalescence was marked by diminished loquacity, and sedateness. There were strikingly exceptional instances. Usually, there was nothing like mirthfulness in the mental manifestations, and a *smile* was frequently noted as one of the harbingers of convalescence. In the case of a female patient, it is noted that she showed no reluctance to exposure of the abdomen, to look for the eruption, etc., until the time of convalescence, when the usual modesty of the sex was resumed.

In conclusion, although the mental characters, aside from delirium, present diversities peculiar to individual cases, those belonging to the majority of cases, may be summed up as follows: sluggishness of all the mental powers; disinclination to exertion, either of the attention, or the will; absence of emotional sensibility, of anxiety as to the issue of the disease, etc. And as a general remark, these traits were prominent, and early displayed, in proportion to the severity of the disease.

*Sleep.* More or less somnolency existed in a large proportion of the cases under analysis. By this I mean, that during the day time, and, in the hospital cases, especially at the time of the daily record, the patient was dozing, and exhibited a marked tendency to sleep. The degree of apparent drowsiness differed considerably in different cases. Generally, the sleep was not profound, nor complete, the patient lying in a state of semi-somnolency, with his eyes closed, readily roused on being addressed, but relapsing shortly into the same state. This condition is peculiar, and in a high degree characteristic of Continued Fever, being seldom observed in other affections, constituting the symptom known as *coma-vigil.*

Somnolency was not uniformly present, but it was much more frequent in the cases of *Typhus*, than in those of the *Typhoid* type. Out of *eleven* cases of *Typhus* in which the records contain information on this point, it was absent in but a single case; while out of *twenty-one* cases of *Typhoid*, it appears not to have been present in *nine.* Hence, it would seem that the peculiar morbid condition of the nervous system upon which this symptom depends, exists almost constantly in *Typhus*, while it only exists in a little more than one-half the cases of *Typhoid.*

Of the cases of *doubtful type*, it existed in *three*, and was absent in *two*, information on this point being deficient in *three.*

In order to ascertain whether any connection existed between this symptom and the issue of the disease, I will give the facts in the fatal cases.

In the *four* cases in *private practice*, which ended fatally, the conditions were respectively as follows:—(*Typhoid.*) In *one* case, vigilance throughout the whole career of the disease, with active delirium; sleep rather heavy, when it did occur; in *one* case, somnolency, the patient being easily roused, until toward the close of life, when it became more and more difficult, and finally impossible; in *one* case, the record is defective on this point. (*Typhus.*) In the fatal case of this type, the patient was somnolent, but easily roused, up to the close of life.

In the *hospital* cases, as follows:—(*Typhoid.*) Vigilance throughout the disease in *one* case; somnolent toward the close of life, but easily roused, in *one* case; somnolent, and roused to only partial intelligence, in *one* case; part of the time somnolent, but easily roused, in *one* case. (*Typhus.*) Somnolent, but easily roused, in *one* case, up to the close of life; somnolent at first, and easily roused, but became insensible, and died in that state, in *one* case. In the fatal case of *doubtful type*, somnolency did not exist.

It would seem that an unfavorable prognosis is in no wise to be based on the presence of this symptom, when it is considered that it does not uniformly exist in the cases which prove fatal; nor, when it does exist, is it uniformly in a marked degree; and when it is also considered that it exists in a large proportion of the cases which terminate in recovery.

It is worthy of remark, that the somnolency of Continued Fever does not often eventuate in insensibility, or coma. In but *two* cases of Typhus, and

*one* case of *Typhoid*, do the histories show such a tendency. A practical consideration connected with this fact is, that the presence of this symptom need not deter us from prescribing anodyne remedies, or opiates, with the hope of substituting for the somnolency peculiar to fever, more complete, and refreshing sleep. If the somnolent condition of a fever patient denoted a comatose tendency, the use of such remedies might be expected to favor such a tendency, and would, therefore, rationally, be injudicious. Experience, probably, will be found to sustain the conclusion, that, under these circumstances, these remedies may be administered with impunity, if not with advantage.

*Coma*. This term is here employed to designate a degree of stupor from which the patient could be but partially roused, or, with great difficulty roused, or, in some instances, a state of complete insensibility; and to these conditions occurring during the career of the fever prior to a few hours before death. As occurring shortly before death, they are more properly considered under the head of *mode of dying*, in which connection they will be referred to hereafter.

Considered under these limitations, *Coma* occurred in but a very small number of the cases analyzed, viz., in *two* cases of *Typhus*, and in *two* of *Typhoid*. In *three* of these cases, the disease proved fatal. In the case in which recovery took place, the comatose condition was accompanied by loud, stertorous respiration, constituting what may be termed *apoplectic coma*. As this event is of rare occurrence in fever, and one which cannot but be regarded as rendering the situation of the patient imminently critical, some farther account of this case may be interesting.

William McDonald, Irish, laborer, single, aet. 27, entered hospital June 6th, 1849.

The person who brought him to the hospital, stated that he had been ill ten days, and that he had arrived in this country about a month previous. The patient was incompetent to give a history of the symptoms; said he did not know how long he had been ill.

7th inst. *Present Symptoms.* He lies most of the time in a dozing state, but is easily roused. Eyes are not suffused. Aspect stupid. Face presents slight dingy redness. Manifests aberration of mind by incoherent replies to questions. Does not mutter. Appears not greatly prostrated. Lies on his back, without changing his position, but moves his upper extremities frequently. Takes some notice of persons around him. Respirations 32. Sibilant *rale* through nares. *Has not been observed to cough.* Tongue furred, quite dry in the centre, moist at sides. Skin moist, and temperature somewhat increased. Pulse 120, well developed. Has had two dejections since his entrance on yesterday. Has urinated freely. Abdomen and chest thickly covered with a faint, dusky eruption, which seems either to have faded, or to be not fully developed. Abdomen soft; no tenderness on pressure. Gurgling in right iliac region. Has taken, since his entrance, Sulph. Morphiæ, gr. 1-8, once.

*Treatment:* S. Morphiæ, gr. 1-6; Tart. Ant. et Pot., gr. 1-4; every *six* hours. Weak milk porridge for diet.

8th. This patient remained yesterday without any notable variation in symptoms, until 8, P. M. It was then observed that he was more somnolent. Pulse 140. Respirations 36, and labored. At 10, P. M., the respirations became stertorous, but he was still roused without difficulty. The stertor continued at intervals through the night. I visited the patient at 6 o'clock this morning. The respiration was then loudly stertorous. Pulse 146, and well developed. Skin hot. He could be roused, with difficulty, so as to open his eyes, and to endeavor to protrude his tongue, but the latter effort was ineffectual. When he opened his eyes, the right upper lid dropped considerably. He retains sufficient muscular strength to raise himself into a sitting posture, and to change his position in bed. He passed urine last night, and had three dejections in bed, unconsciously. Has not vomited. I directed the hair to be cut close to the scalp, the ice-cap to be applied, the head elevated, sinapism to the neck; (it had already been applied to the feet and hands.) Tart. Ant. et Pot., gr. ss., to be repeated in twenty minutes.

1-4 before 8, A. M.; a manifest improvement in symptoms. The stertor is nearly gone. Lies with his eyes open, and takes some notice. Protrudes partially his tongue, on being requested. Pulse diminished in frequency, and, notably, in volume and force. Skin moist. No nausea. Antimony to be continued in doses of gr. 1-4, and omitted, if nausea occur. Ice to head continued, and warm applications to extremities.

11 A. M. Patient again stertorous, but can be partially roused. Antimony discontinued. Blister, 6x4, to nucha.

At 4, P. M., symptoms are not given, but the following prescription is recorded: Iodid. Potassii, grs. v, every two hours.

9th. Stertorous respiration continued during the night, at some times increased, and at some times diminished. Since 4, A. M., respiration has been free from stertor, and tranquil, except that the expiration is almost constantly attended by a groan. He is apparently conscious this morning, but does not speak He moves his arms when requested, and as requested, but slowly, and with difficulty. He partially protrudes his tongue, which is covered with a thick moist coating. Pulse is 120. Had free dejections in bed during night. Has urinated freely. He lies with his eyes open, and appears to take notice. 'Pupils not dilated.

He has taken Iodid. Potassii, grs. v, every two hours, and ice has been almost constantly applied to the head.

Treatment for to-day as follows:—Iodide of Potassium, grs. v, every four hours. Proto-chlor. Hydrarg. gr. 1, hourly. Continue ice-cap. Essence of beef has been given frequently, and is to be continued.

10th. Passed a restless night, but without stertor. Had one dejection in bed. Takes notice of persons around him. Respiration now easy and tranquil. Protrudes his tongue, and essays to speak, but cannot enunciate distinctly. Tongue covered with a thin white coating. He appears to comprehend whatever is said to him.

*Treatment:* Sulph. Quiniæ, grs. ij, every four hours. Cont. essence of beef.

P. M. Has had three dejections. They occurred in bed, but he has cognizance of them, and endeavors to get out of bed at that time. No stertor. Lies awake; urinates freely. Pulse (figures not legible) well developed.

Omit Quinia, and give. *per enema,* Tinct. opii, ʒj. Apply blisters behind ears. Sptts, ether nitrosus, ʒj, every four hours. Omit essence of beef.

11th. Rested well part of the night, and part of the time was restless. This morning, aspect more intelligent, appears to comprehend all that is said to him, but cannot control the muscles sufficiently to enunciate distinctly. Respirations 40; skin hot. Pulse 120, well developed. Tongue dry at tip. One dejection this morning, pretty large. Got up to defecate. Urinates freely. Blisters have vesicated well. Ice is still applied to the head. Has some carphologia.

*Treatment:* Sup. Tart. Pot., ℥ss. Nit. Pot., ʒij. Aquæ, 0j. To be drank during the day.

12th. Restless early part of night, but after taking Tart. Ant. and Pot., gr. $\frac{1}{4}$, became more quiet. This morning quite as comfortable as yesterday. He is now sitting in defecating chair. Tongue cleaning. Manifests more intelligence, and can enunciate better than yesterday. Four dejections since yesterday morning. Secretion of urine abundant.

*Treatment:* P. Doveri, grs. iij. Tart. Ant. and Pot., gr. $\frac{1}{8}$, every four hours. Omit Sol. Sup. Tart. and Nit. Pot.

13th. Aspect better. Tongue improving. One dejection. Skin less hot. Pulse 108. Respirations tranquil, and 32.

*Treatment:* Tart. Ant. and Pot., gr. $\frac{1}{8}$, every four hours.

14th. Had an uncomfortable night. Respirations accelerated and labored. *Cough troublesome.* Aspect as good as yesterday. Seems bright; understands what is said, and raises himself briskly in bed. Respirations 30; expiration attended by a sibilant nasal rale. Pulse, 120, well developed. No dejection. Urinates freely. Tongue moist and cleaning. Continues to enunciate imperfectly.

Cont. Treatment.

15th. Good night. Aspect improved. Pulse 108, less developed. Skin perspiring. Tongue cleaning. Respirations 36, and not labored. Enunciates better. No dejection for 48 hours. *Cough has become a troublesome symptom. Expectorates considerable puruloid looking matter.*

16th. Restless night. Respirations short, frequent, and labored. Has expectorated considerable muco-purulent matter. Skin warm and moist. Pulse 112. Had large dejection this morning, moulded, and natural in appearance. Respirations 24, and now easy.

*Treatment:* Carb. ammoniæ, and Tart. Ant. and Pot., if respirations become accelerated or labored.

17th. Aspect better. Comfortable night. Skin cool. Pulse 100. Copious muco-purulent expectoration continues. Respirations easy.

18th. Symptoms continue the same. Distinct relative dullness on percussion over the left side, posteriorly, at the inferior angle of the scapula, with a loud mucous rale; on right side, posteriorly, sonorous rale. He continued to improve, but exhibited some delirium until the 25th, when it is noted that the pulse were 140, temperature of surface increased, copious purulent expectoration continuing, and respirations 32.

27th. Aspect and symptoms improved, but still some delirium at night; copious purulent expectoration. Pulse 108. Skin cool, and perspiring. Chest dull on percussion on the left side, laterally and posteriorly, and anteriorly at inferior third. Respiration over dull portions, tubular. No rales perceived.

On the 29th, he was up and dressed.

July 1st, he was walking about, and from this date, he convalesced rapidly.

*Remarks.* The history of this case is given in detail, on account of the occurrence of coma, with apoplectic stertor, and the recurrence of this condition, in a somewhat paroxysmal manner. The investigation of the case, I regret to say, as is now apparent, was incomplete, at the time these symptoms became developed. The chest should have been examined at that time, for, although no cough was noticed anterior to, or or in connection with the coma, it is not improbable that physical exploration would then have revealed the existence of pneumonitis, the existence of which, subsequently, was apparent. The occurrence of a muco-purulent expectoration, which became at once a prominent symptom, together with cough, is presumptive evidence that pneumonic inflammation had been present for some time, for it would not be expected that a purulent formation would have accompanied the inflammatory attack from its very onset. Assuming that pneumonitis existed, and its characteristic symptoms masked by those appertaining to the brain, it becomes an interesting question whether the cerebral affection may not have been, in a measure, dependent upon the pneumonic inflammation. It would be improper to discuss this question, inasmuch as the data must be assumed.

In this pathological aspect, the case is one of those which enforces the importance of physical exploration of the chest, under circumstances in which pulmonary disease may be present, with an absence of rational symptoms directing inquiry in that direction, the attention, as in this instance, being absorbed with prominent cerebral symptoms, which, as is well known, occasionally mask affections of other organs.

(*To be Continued.*)

